# Supplementary material for: Imaging the kinetics of anisotropic dissolution of bimetallic core–shell nanocubes using graphene liquid cells
Source: Nat Commun. 2020 Jun 16;11:3041. doi: 10.1038/s41467-020-16645-3 (PMC7297726; doi:10.1038/s41467-020-16645-3)
Supplement: Supplementary file 1 — Supplementary Information [file 41467_2020_16645_MOESM1_ESM.pdf]

Supplementary Information

# Imaging the kinetics of anisotropic dissolution of bimetallic core-shell nanocubes using graphene liquid cells

Chen et al.

## Supplementary Figures

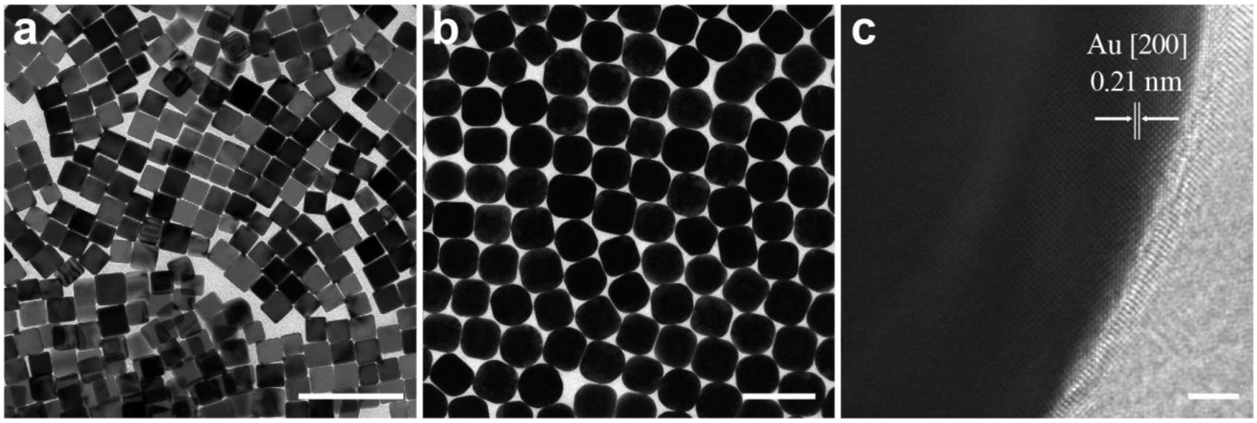


**Supplementary Figure 1 | TEM characterization of Pd@Au nanocubes.** Additional TEM images of (a) Pd nanocubes (edge length: 44.1 ± 3.9 nm) and (b) Pd@Au core-shell nanocubes (Au shell thickness: 35.3 ± 4.3 nm). (c) HRTEM image acquired near the corner region of a Pd@Au nanocube. Scale bars: (a,b) 200 nm, (c) 2 nm.

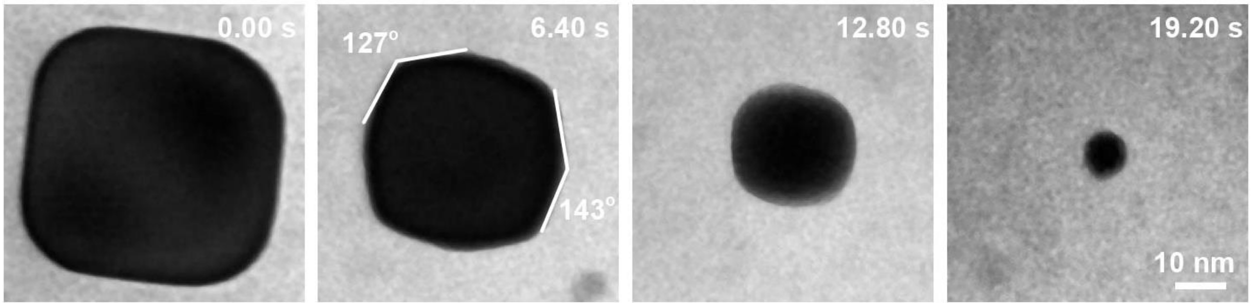


**Supplementary Figure 2 | Dissolution kinetics of Au nanocubes.** Time-lapse TEM images extracted from Supplementary Movie 5.

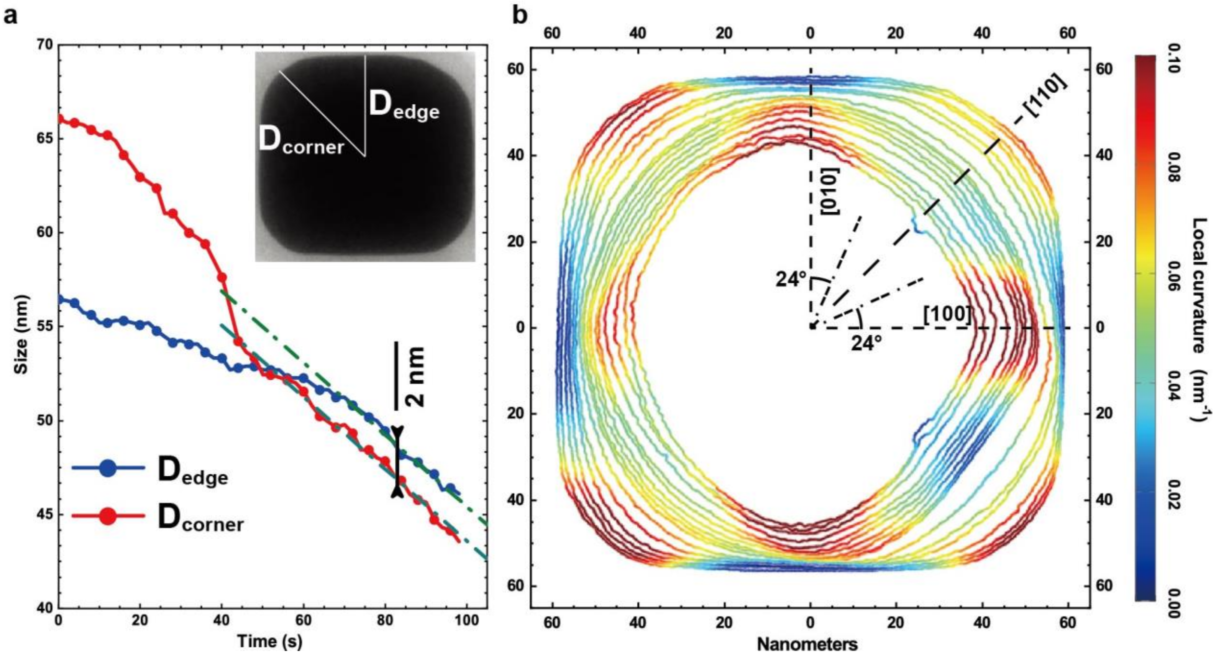


**Supplementary Figure 3 | Dissolution kinetics and transformation pathway of a single Pd@Au nanocube.** (a) Plots of linear dimensions $D_{corner}$ and $D_{edge}$ as a function of time for a dissolving Pd@Au nanocube (44-nm core and 35-nm shell). (b) Time-domain contour plot showing the morphological transformation of a dissolving Pd@Au nanocube prior to exposure of the Pd core. Individual contour lines are color-coded according to local curvature and are spaced in time by 2.0 s.

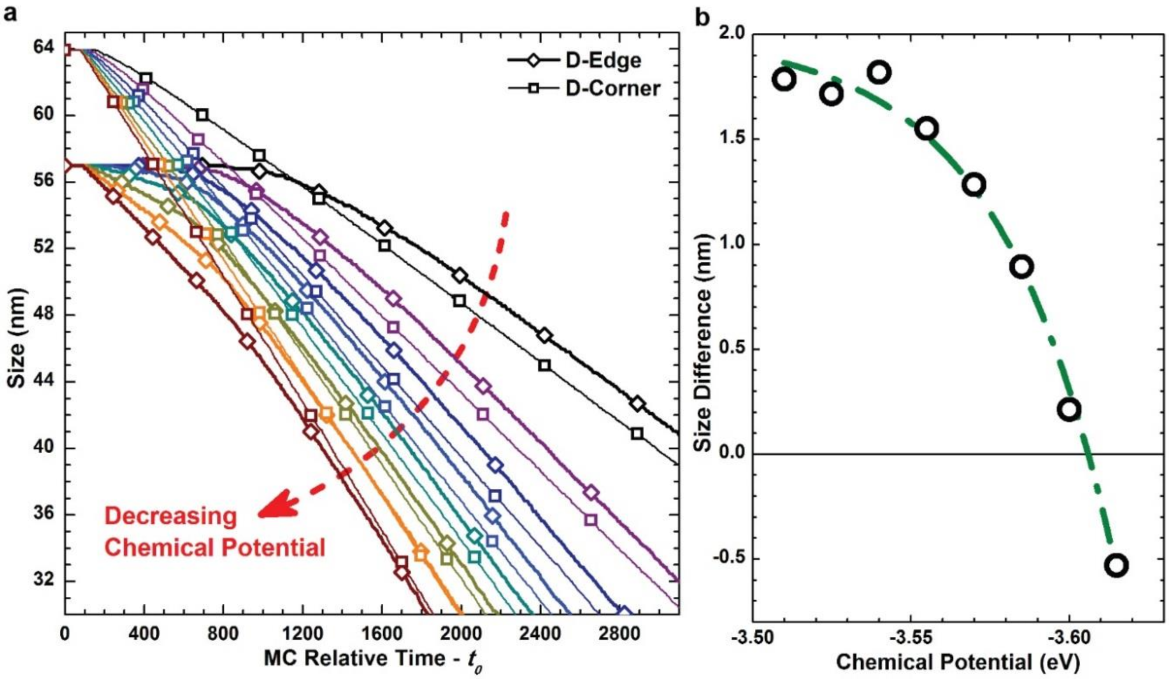


**Supplementary Figure 4 | Dissolution kinetics of Pd@Au nanocube from MC simulations.** (a) Evolution of the linear dimensions $D_{corner}$ and $D_{edge}$ for a dissolving Pd@Au nanocube (44-nm core and 35-nm shell). (b) Intermediate equilibrium dimension difference $D_{edge} - D_{corner}$ as a function of chemical potential of the oxidative environment. A more negative chemical potential corresponds to a more oxidizing environment.

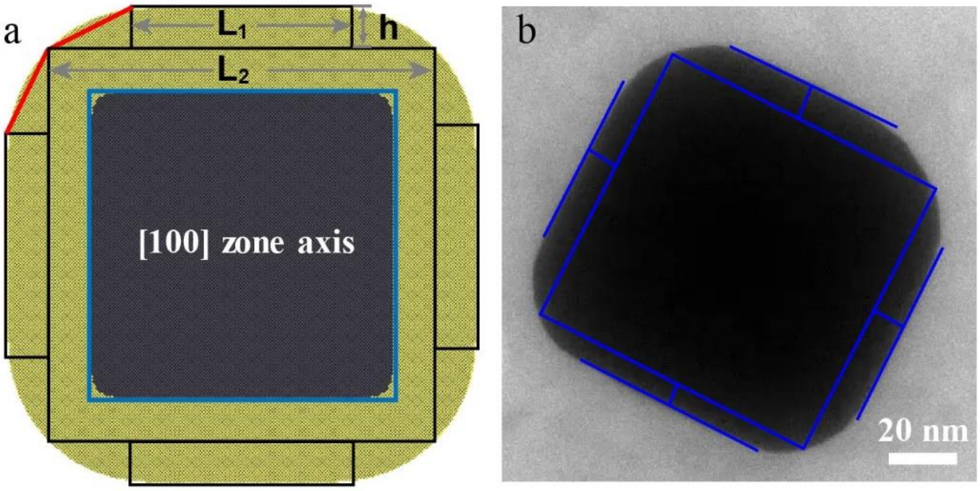


**Supplementary Figure 5 | Volume estimation of dissolving Pd@Au nanocubes.** (a) Geometrical model used for volume estimation. (b) Example of frame overlay for partially etched Pd@Au nanocubes. The total volume is calculated as $\mathbf{V} = L_2^3 + 2h(L_2^3 - L_1^3)/(L_2 - L_1)$.

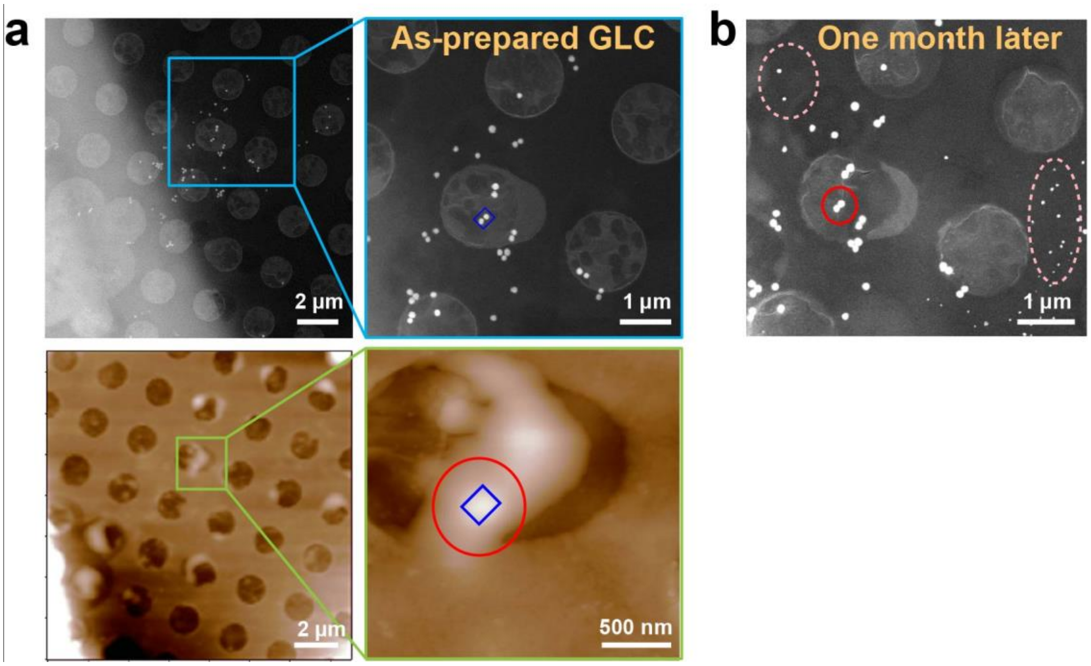

**Supplementary Figure 6 | Correlative SEM-AFM images of GLC pockets.** (a) SEM (top) and AFM (bottom) images of as-prepared GLCs encapsulating Pd@Au nanocubes. (b) SEM image of the same GLC area acquired one month after its preparation. The particles highlighted by dotted ellipsoids are believed to be newly formed during GLC storage, whereas several original particles appear to be either displaced from its original position or have grown larger, which further confirms the presence and the integrity of liquid pockets.

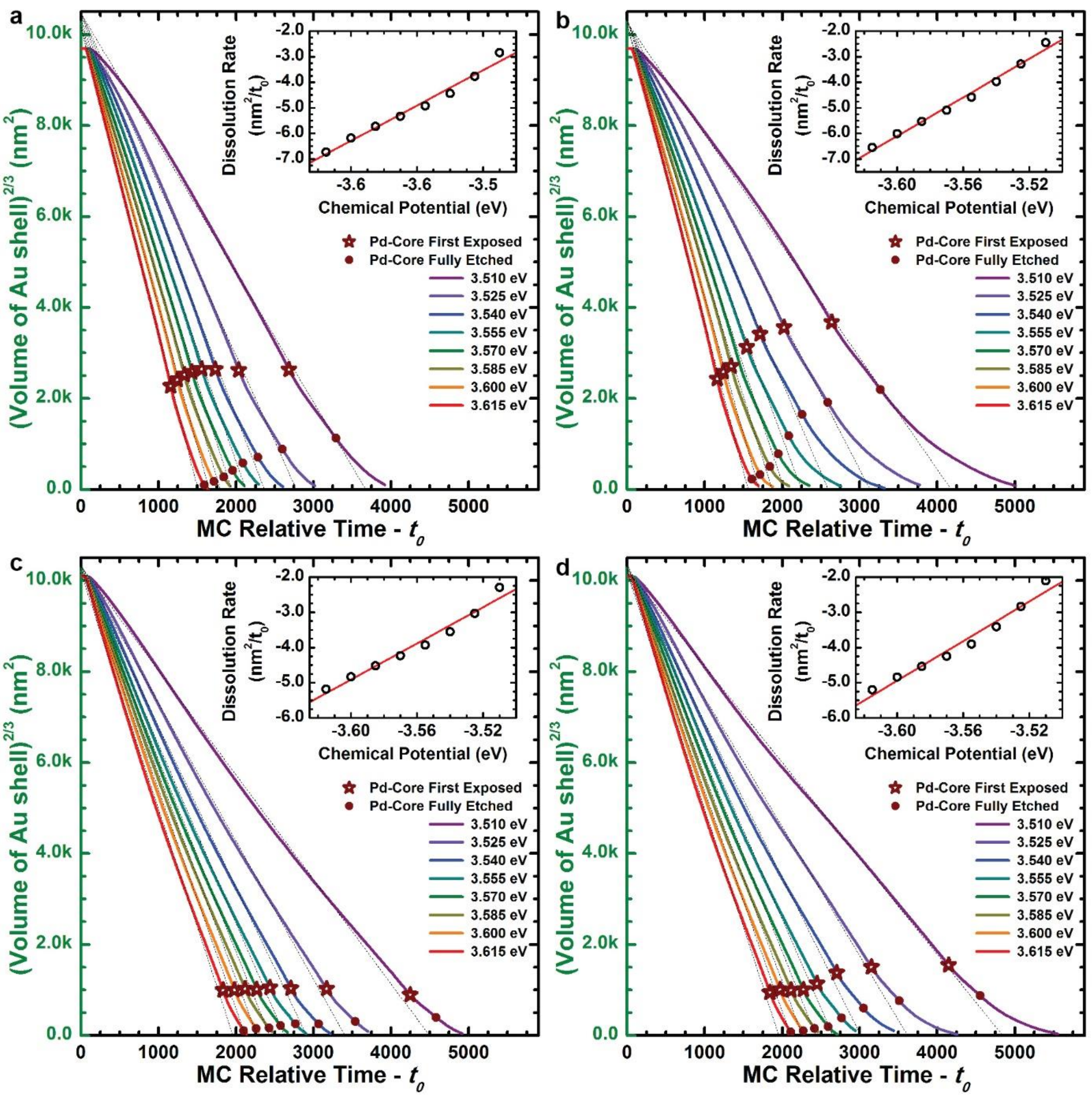


**Supplementary Figure 7 | Dissolution kinetics of the Au shell for Pd@Au core-shell nanocubes at different strength of oxidative environment**. Dissolution of (a,b) thin-shell Pd@Au (70-nm core and 22-nm shell) and (c,d) thick-shell Pd@Au (44-nm core and 35-nm shell) nanocubes at chemical potentials ranging from -3.510 eV to -3.615 eV. Results are shown for absence (a,c) and inclusion (b,d) of graphene as the top and bottom encapsulating sheets. Insets show the dissolution rate of the Au shell as a function of chemical potential before the exposure of the Pd core.

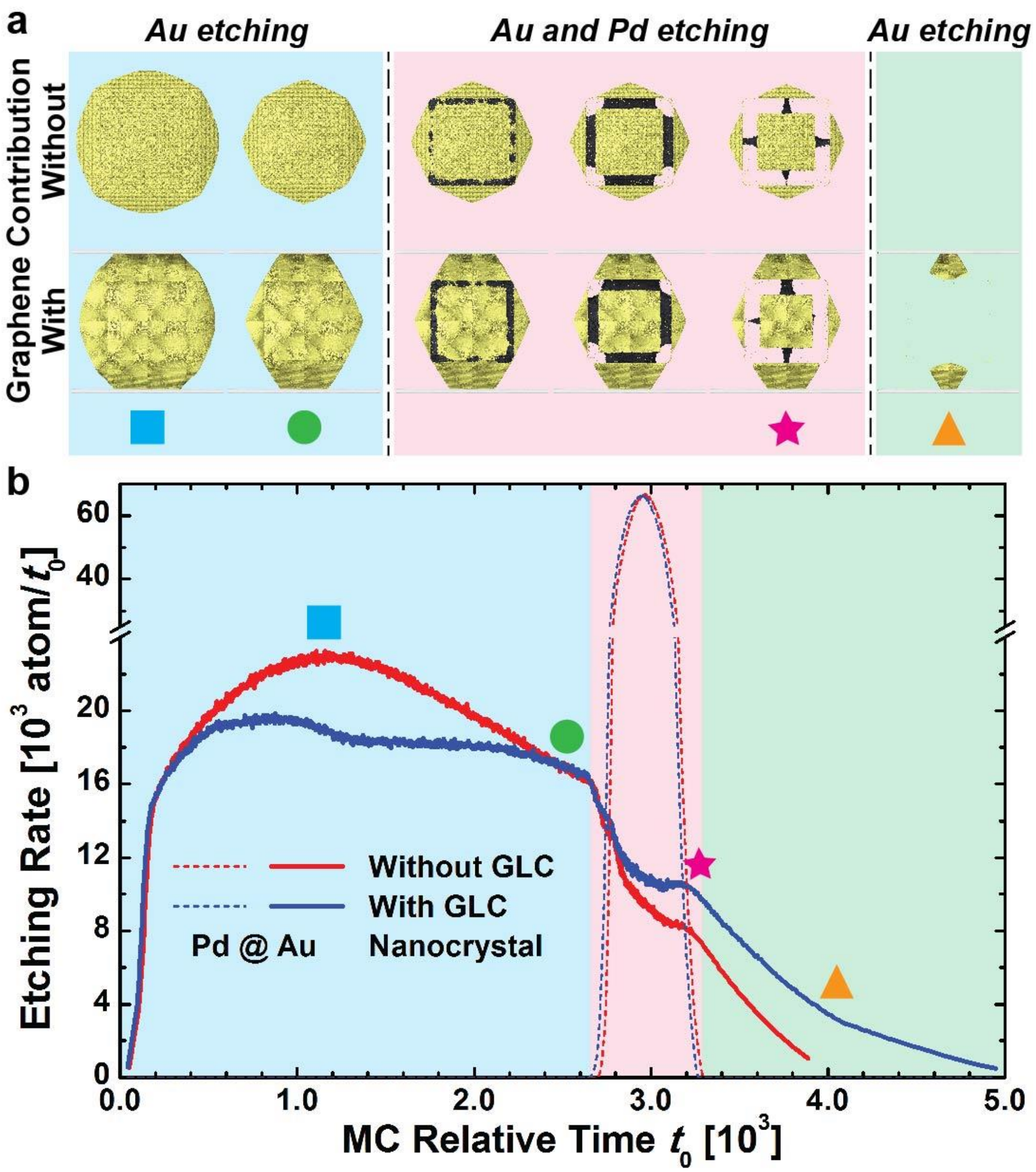


**Supplementary Figure 8 | Anisotropy of chemical etching induced by graphene windows.** (a) Snapshots of intermediates and (b) etching rate (rate of atoms removal) as a function of the Monte Carlo relative reaction time of a nanocube (70-nm Pd core and 22-nm Au shell) exposed to a weakly oxidative environment (chemical potential -3.510 eV) isolated (red, top time series) and trapped by the graphene window (blue, bottom time series).

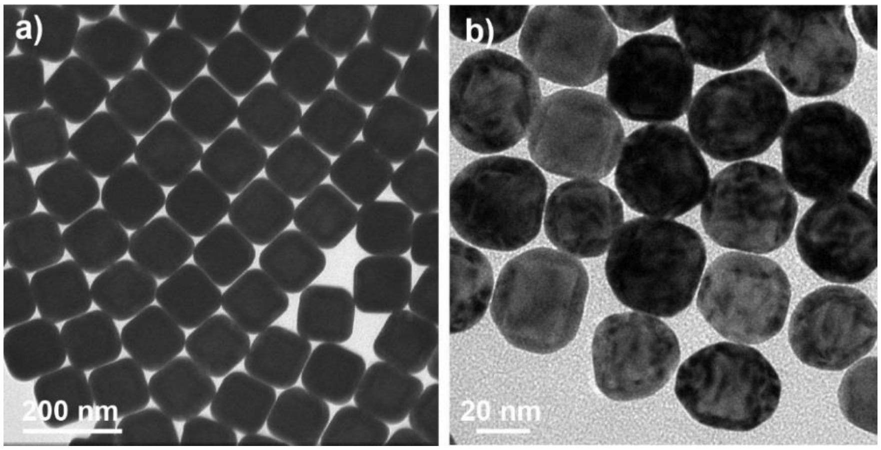


**Supplementary Figure 9 | TEM characterization of Pd@Au nanocubes.** (a) TEM image of Pd@Au nanocubes (70-nm core and 22-nm shell). (b) TEM image of Pd@Au nanocubes (25-nm core and 5-nm shell).

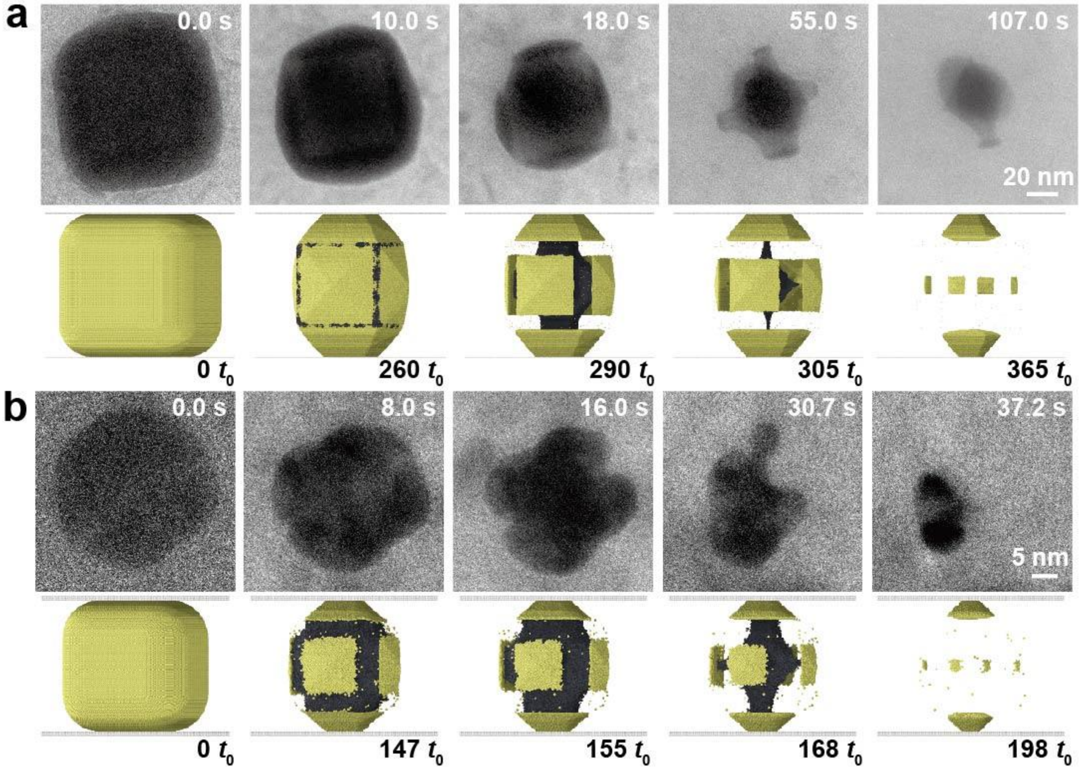


**Supplementary Figure 10 | Complex shape intermediates generated from etching of bimetallic core-shell cubes.** (a, b) Time-lapse TEM images and corresponding snapshots from Monte Carlo simulations extracted from (a) Supplementary Movies 7b and 4 on the dissolution of a thin-shell Pd@Au nanocube (70-nm core and 22-nm shell) under a weakly oxidizing environment (chemical potential used in simulation: -3.525 eV) and (b) Supplementary Movies 8 and 9 on the dissolution of a small Pd@Au nanocube (25-nm core and 5-nm shell) under an intermediate oxidizing strength (chemical potential in simulation: -3.555 eV).

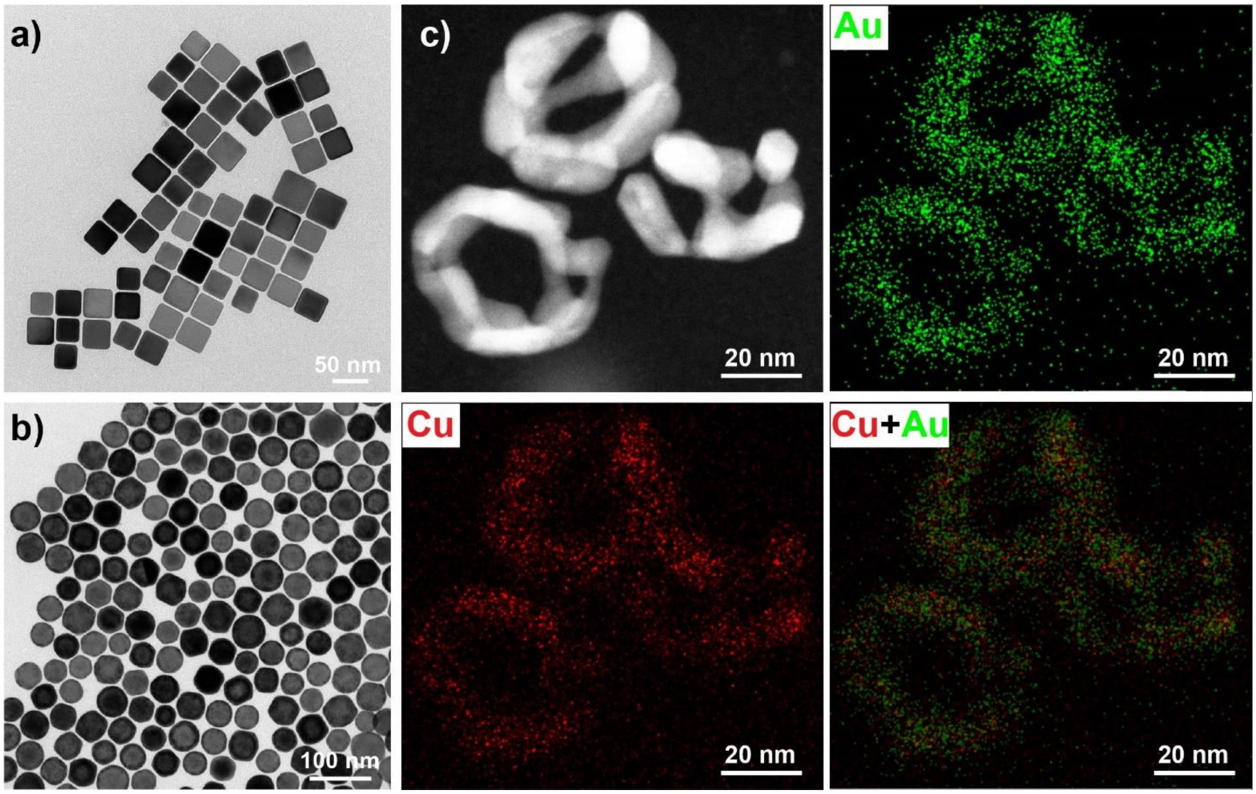


**Supplementary Figure 11 | TEM characterization of Cu@Au nanocubes.** (a) TEM image of Cu nanocubes (edge length: 44.3 ± 3.1 nm**).** (b) TEM image of Cu@Au nanocubes (shell thickness: 2.6 ± 0.4 nm**).** (c) STEM-EDX elemental mapping of Au nanocages resulting from etching of Cu@Au nanocubes. The GLCs became dried out shortly after etching started, making it suitable for subsequent STEM-EDX mapping as further etching during STEM scans is inhibited. The Cu signal observed on STEM-EDX maps is likely attributed to precipitated Cu salts as a result of sample dryout.

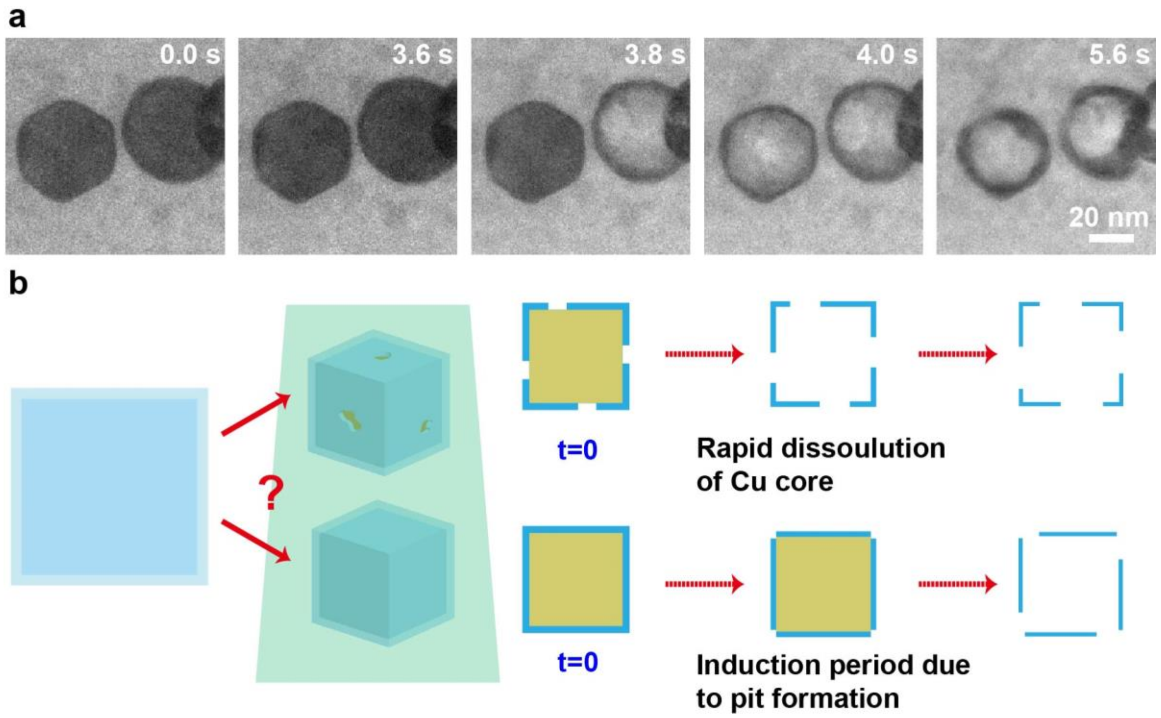


**Supplementary Figure 12 | Simultaneous imaging of dissolution of multiple Cu@Au nanocubes** (a) Time-lapse TEM images extracted from Supplementary Movie 10b showing the dissolution of a pair of Cu@Au nanocubes. (b) Schematic illustration of a potential application of *in-situ* GLC imaging to evaluate shell thickness and uniformity within an ensemble of core-shell nanoparticles.

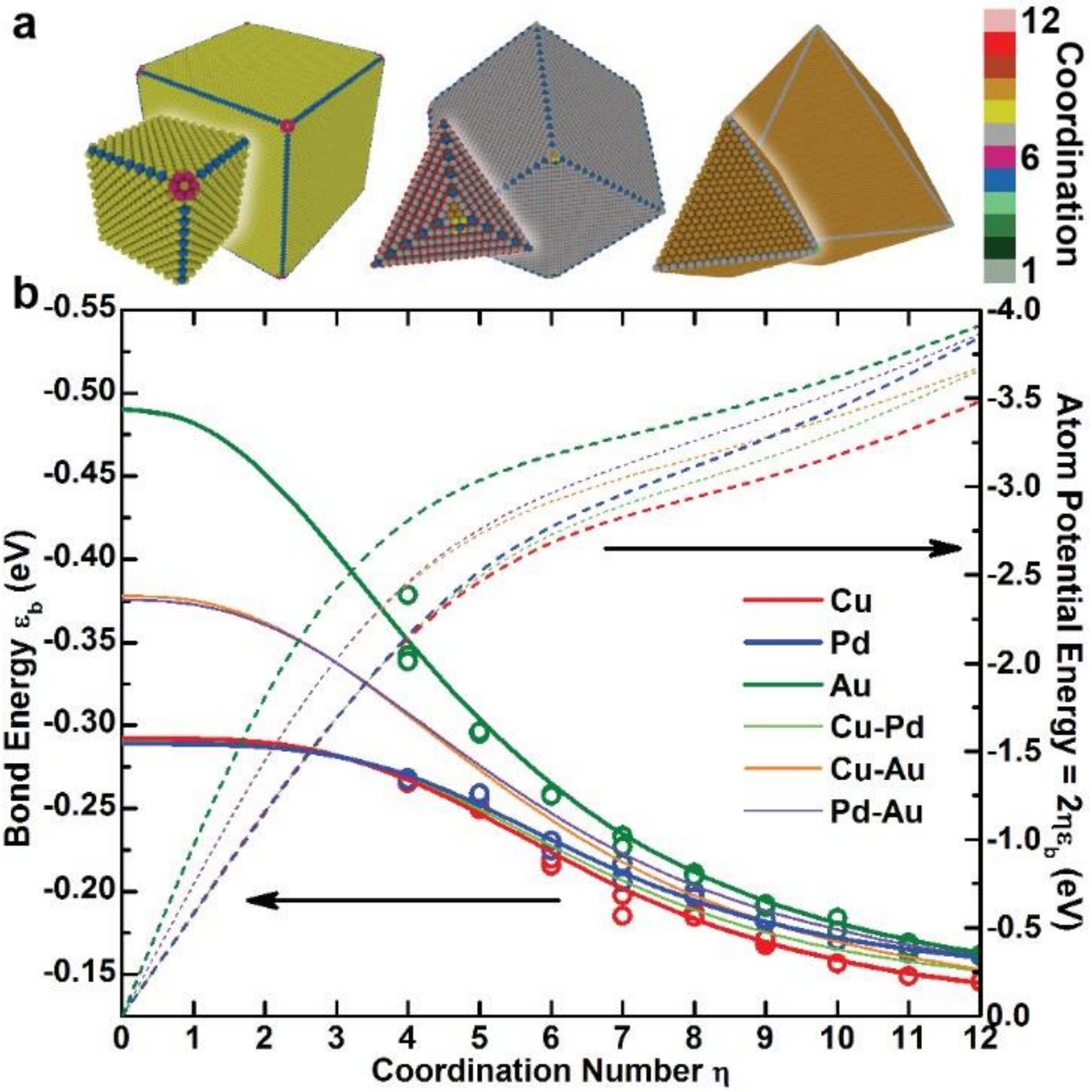


**Supplementary Figure 13 | Bond energy as a function of atomic coordination number.** (a) Coordination number for Pd nanocrystals with three different shapes: cube, rhombic dodecahedron, octahedron. Inset: Zoom-in at the corners. Nanocrystal shape and atom coordination differ from ideal because evaluated after molecular dynamics equilibration of the atomistic models. (b) Calculated average bond energy as a function of coordination number (open dots) and their regression profile (continuous line). Parameters of the regression profiles are shown in Table S1. The per atom potential energy profiles are also shown as reference (dashed line). Bond energies for bimetallic systems were computed by the Berthelot mixing rule.

# Supplementary Tables

**Supplementary Table 1 | Simulation parameters.** Parameters for the logistic functions ($\epsilon_b = (A_1 - A_2)/(1 + (\eta/\eta_0)^p) + A_2$) approximating the bond energies in Supplementary Figure 13 and characteristic standard deviation (SD) of the measured values.

| **Bond** | $\eta_0$ | $p$ | $A_1$ | $A_2$ | SD |
|---|---|---|---|---|---|
| **Cu/Cu** | 0.24946 | 3.38346 | 0.29222 | 0.12473 | 0.002680 |
| **Pd/Pd** | 6.71290 | 3.61737 | 0.28883 | 0.14467 | 0.003070 |
| **Au/Au** | 4.92865 | 2.36958 | 0.49022 | 0.12329 | 0.003140 |
| **Cu/Pd** | 6.70079 | 3.48849 | 0.29051 | 0.13443 | 0.002868 |
| **Cu/Au** | 5.82222 | 2.56365 | 0.37793 | 0.11789 | 0.002901 |
| **Pd/Au** | 5.82903 | 2.57729 | 0.37561 | 0.12753 | 0.003105 |
