# Supplementary material for: Imaging the kinetics of anisotropic dissolution of bimetallic core–shell nanocubes using graphene liquid cells
Source: Nat Commun. 2020 Jun 16;11:3041. doi: 10.1038/s41467-020-16645-3 (PMC7297726; doi:10.1038/s41467-020-16645-3)
Supplement: Supplementary file 3 — Description of Additional Supplementary Files [file 41467_2020_16645_MOESM3_ESM.pdf]

Description of Additional Supplementary Files

File Name: Supplementary Movie 1
Description: TEM movie of dissolution of a Pd@Au core-shell nanocube (44-nm core and 35-nm shell) without (a) and with (b) the graphene liquid cell sample tilted around the y-axis by 27° before dissolution was initiated. The y-axis makes an angle of about 45 degrees with the horizontal axis of the phosphor screen. The electron dose rate was 617 electrons/Å^2·s.

File Name: Supplementary Movie 2
Description: (a) TEM movie of slow dissolution of a Pd@Au core-shell nanocube (44-nm core and 35-nm shell). The electron dose rate was 254 electrons/Å^2·s. (b) The same movie with overlaid contour plots that are color-coded according to particle local curvature.

File Name: Supplementary Movie 3
Description: TEM movie showing the step recession process occurring on {hk0}-type vicinal facets, generating a pyramid from an initially flat (100) facet of the Au shell. The electron dose rate was 254 electrons/Å^2·s.

File Name: Supplementary Movie 4
Description: Kinetic Monte Carlo simulations of Pd@Au core-shell nanocubes with the same edge length of 112 nm but different core-to-shell volume ratios etched at different chemical potentials. Simulations were performed by taking into account the effects of graphene window as well as for nanocrystals in an isotropic environment (i.e. without graphene windows). Central-cut images are shown with the nanocrystal viewed perpendicular to the TEM viewing direction. Note: Graphene sheets are not rendered.

File Name: Supplementary Movie 5
Description: TEM movie of dissolution of a monometallic Au nanocube. The electron dose rate was 254 electrons/Å^2·s.

File Name: Supplementary Movie 6
Description: TEM movie of dissolution of a Pd@Au core-shell nanocube (70-nm core and 22-nm shell) producing a Au-Pd-Au segmental nanorod. The electron dose rate was 254 electrons/Å^2·s.

File Name: Supplementary Movie 7
Description: (a) TEM movie of rapid dissolution of a Pd@Au core-shell nanocube (70-nm core and 22-nm shell). The electron dose rate was 1055 electrons/Å^2·s. (b) TEM movie of slow dissolution of a Pd@Au core-shell nanocube (70-nm core and 22-nm shell). The electron dose rate was 254 electrons/Å^2·s.

File Name: Supplementary Movie 8
Description: TEM movie of dissolution of a small Pd@Au core-shell nanocube (25-nm core and 5-nm shell). The electron dose rate was 254 electrons/Å^2·s.

File Name: Supplementary Movie 9

Description: Kinetic Monte Carlo simulations of Pd@Au core-shell nanocubes (25-nm core and 5-nm shell) etched at different chemical potentials. Simulations were performed by taking into account the effects of top and bottom graphene windows as well as for nanocrystals in an isotropic environment (i.e. without graphene windows). Central-cut cross-sectional images are shown with the nanocrystal viewed perpendicular to the TEM viewing direction. Note: Graphene sheets are not rendered.

File Name: Supplementary Movie 10
Description: TEM movies of dissolution of (a) a single and (b) a pair of Cu@Au core-shell nanocubes (44.3-nm core and 2.6-nm shell). The electron dose rate was 254 electrons/$Å^2 \cdot s$.

File Name: Supplementary Movie 11
Description: Kinetic Monte Carlo simulations of Cu@Au core-shell nanocubes (44-nm core and 3-nm shell) etched at different chemical potentials. Simulations were performed by taking into account the effects of graphene window as well as for nanocrystals in an isotropic environment (i.e. without graphene windows). Central-cut images are shown with the nanocrystal viewed perpendicular to the TEM viewing direction. Note: Graphene sheets are not rendered.
